# Comparative Proteomics Highlights that GenX Exposure Leads to Metabolic Defects and Inflammation in Astrocytes

**DOI:** 10.1021/acs.est.4c05472

**Published:** 2024-11-05

**Authors:** Abdulla Abu-Salah, Müberra Fatma Cesur, Aiesha Anchan, Muhammet Ay, Monica R. Langley, Ahmed Shah, Pablo Reina-Gonzalez, Rachel Strazdins, Tunahan Çakır, Souvarish Sarkar

**Affiliations:** † Department of Environmental Medicine, University of Rochester Medical Center, 575 Elmwood Avenue, Rochester, New York 14620, United States; ‡ Department of Bioengineering, Gebze Technical University, Gebze, KOCAELİ 41400, Turkey; § Department of Neuroscience, University of Rochester Medical Center, 575 Elmwood Avenue, Rochester, New York 14620, United States; ∥ Department of Molecular Pharmacology & Experimental Therapeutics, Department of Neurology, Department of Physical Medicine & Rehabilitation, Mayo Clinic, Gonda Building, 19th Floor, 200 First St. SW, Rochester, Minnesota 55905, United States

**Keywords:** behavior, HFPO-DA, nontargeted proteomics, astrocytes, Drosophila

## Abstract

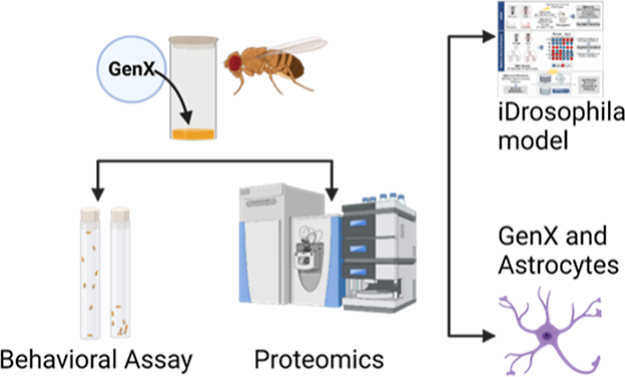

Exposure to PFAS such as GenX (HFPO dimer acid) has become increasingly common due to the replacement of older generation PFAS in manufacturing processes. While neurodegenerative and developmental effects of legacy PFAS exposure have been studied in depth, there is a limited understanding specific to the effects of GenX exposure. To investigate the effects of GenX exposure, we exposed *Drosophila melanogaster* to GenX and assessed the motor behavior and performed quantitative proteomics of fly brains to identify molecular changes in the brain. Additionally, metabolic network-based analysis using the *i*Drosophila1 model unveiled a potential link between GenX exposure and neurodegeneration. Since legacy PFAS exposure has been linked to Parkinson’s disease (PD), we compared the proteome data sets between GenX-exposed flies and a fly model of PD expressing human α-synuclein. Considering the proteomic data- and network-based analyses that revealed GenX may be regulating GABA-associated pathways and the immune system, we next explored the effects of GenX on astrocytes, as astrocytes in the brain can regulate GABA. An array of assays demonstrated GenX exposure may lead to mitochondrial dysfunction and neuroinflammatory response in astrocytes, possibly linking non-cell autonomous neurodegeneration to the motor deficits associated with GenX exposure.

## Abstract 2

The effect of water contamination of second generation PFAS’s like GenX has been understudied in the neurodegeneration, and this nontargeted proteomic study provides a dataset as well as a potential mechanism through which GenX may regulate brain homeostasis.

## Introduction

Per-and polyfluoroalkyl substances (PFAS) make up a large group of synthetic organic chemicals that are persistent in the environment and are resistant to degradation. PFAS are used in numerous industrial processes and have led to global contamination of drinking water and the environment as persistent environmental contaminants. Here, we are interested in a more modern variation of PFAS–GenX. GenX is a newly regulated PFAS found on nonstick coatings and is a major byproduct of many manufacturing processes.^1^ Many manufacturing companies have switched from older generations of PFAS to GenX due to its shorter half-life in the body, however, there is competing evidence on accumulation, half-life, and further toxicity.^2^

In 2017, GenX was found in a drinking water source in North Carolina, indicating that exposure is not limited to a small group of individuals. Rather, GenX exposure may already encompass a large portion of the population. There is preliminary evidence that PFAS can accumulate in the brain. Studies with cerebrospinal fluid and serum have shown that PFAS is able to reduce the integrity of blood-brain barrier, thus leading to its accumulation.^3^ This phenomenon is seen in many other neurotoxicants such as pesticides, heavy metals, and mixed metal emissions. Since the brain has many quiescent neurons, the persistent accumulation of these long-chain carbon molecules can cause damage to neurons and other cell types in the central nervous system. There is ongoing evidence of accumulation of PFAS in the brains of wildlife surrounding manufacturing plants, with experiments showing increased brain concentration of PFAS as well as developmentally altered neurotransmitters, especially acetylcholine.^4^ Given concerns of bioaccumulation and toxicity in workers and those who are exposed environmentally, the United States Environmental Protection Agency (EPA) has worked with the manufacturing industry to significantly reduce the production of PFAS, PFOA, PFOS, and other related compounds.^5^

There are even fewer studies done on the neurotoxicity of GenX, which makes it increasingly more important to develop an understanding of its neurotoxicity and the molecular pathways that it impacts. Moreover, we are interested in understanding whether the effects of GenX exposure were similar to those of other well-characterized PFAS and whether they would display parallel motor deficits. Omics data-based approaches and network-based analyses have been used to comprehensively characterize the effects of various chemicals on cellular processes.^6,7^ As a network-based approach, genome-scale metabolic network (GMN) modeling can provide a holistic insight into the metabolic alterations in response to the exposure of a chemical compound when integrated with a related omics data set. Together, an integrative approach consisting of computational (omics data and network-based analyses) and experimental strategies may be promising for the comprehensive and accurate investigation of both GenX toxicity and the underlying metabolic changes in the brain.

In this study, we exposed *Drosophila* to GenX ad libitum and performed behavioral assays in a sex specific manner. GenX exposure led to climbing and locomotor deficits in both males and female flies but the changes were more pronounced in female flies. Further, female flies exposed to GenX had a more severe seizure-like phenotype than males. Hence, to understand the molecular signatures that are occurring in female *Drosophila* at an early time point, we performed proteomics. The analysis of proteomics data and the metabolic network model, *i*Drosophila1, offers some insight into the molecular pathways that are affected when chronically exposed to GenX. Since PFAS exposure has been previously linked to PD, we performed a comparative proteomics analysis on a proteomic data set of transgenic *Drosophila*, expressing human α-synuclein (αSyn). The symptomology of PFAS exposure can be similar to Parkinson’s disease (PD) or other synucleinopathies, which are neurodegenerative diseases caused by the impairment of αSyn metabolism resulting in its aggregation. By comparing the GenX data set to the αSyn data set, we have identified glial genes and metabolic pathways that are altered by GenX exposure. Understanding the potential neurotoxic effects of this newer, less-characterized PFAS chemical without any monitoring or enforcement provisions in place in the US for another few years is key to putting forth proper regulations and protecting vulnerable populations. It can also help further identify unique etiologies of PD and other neurodegenerative diseases as that may fall outside of our normal understanding of the causes of neurodegenerative diseases.

## Methods

### Fly Husbandry

All flies were maintained in 25 °C incubator. GenX was mixed in the food of the adult flies, and flies were exposed 2–3 days post eclosion. Since GenX was found not to accumulate in flies, we changed the food every 3 days.^8^ Unlike larvae, adult flies do not consume a lot of food. We have seen with other chemicals that the body burden of chemicals is generally 1000–5000 times less than concentrations in the food. Previous studies have also used similar doses of GenX in flies.^8^ We performed behavioral assays every 3 days for 20 days to identify time points where the flies first started displaying motor behavior deficits. Then the proteomics were performed on flies at a time point that precedes behavioral deficits (5 days).

### *Drosophila* Behavior Assays

#### Climbing and Locomotor Assay

Climbing assays were performed as previously described.^9^ Briefly, flies were transferred to vials without food and acclimatized for 3 min before tapping the flies down and counting the percentage of flies that cross 5 cm in 10 s. Locomotor assay was performed as previously described.^10^

### Proteomics

#### Sample Preparation for Mass Spectrometry

Samples for protein analysis were prepared essentially as previously described.^11,12^ Proteomes were extracted using a buffer containing 200 mM EPPS (pH 8.5), 8 M urea, and protease inhibitors. Following lysis, each sample was reduced with 5 mM TCEP. Cysteine residues were alkylated using 10 mM iodoacetamide for 20 min at RT in the dark. Excess iodoacetamide was quenched with 10 mM DTT. 100 μg portion of each proteome was precipitated and resolubilized in 200 mM EPPS pH 8.5. Samples were digested with Lys-C (1:50) overnight at RT and subsequently with trypsin (1:100) for 6 h at 37 °C. Anhydrous acetonitrile was added to each sample to achieve a final concentration of 33% acetonitrile. 50 μg of peptides from each sample were labeled with TMTPro reagents (Thermo Fisher) for 2 h at room temperature. Labeling reactions were quenched with 0.5% hydroxylamine and acidified with formic acid. Acidified peptides were combined and desalted with Sep-Pak (Waters).

#### Basic pH Reversed-Phase Separation (BPRP)

TMT labeled peptides were solubilized in 5% ACN/10 mM ammonium bicarbonate, pH 8.0, and ∼300 μg of TMT labeled peptides were separated by an Agilent 300 Extend C18 column (3.5 μm particles, 4.6 mm ID and 250 mm in length). An Agilent 1260 binary pump coupled to a photodiode array detector (Thermo Scientific) was used to separate the peptides. A 45 min linear gradient from 10% to 40% acetonitrile in 10 mM ammonium bicarbonate pH 8.0 (flow rate of 0.6 mL/min) separated the peptide mixtures into a total of 96 fractions (36 s). A total of 96 fractions were consolidated into 24 samples in a checkerboard fashion and vacuum-dried to completion. Each sample was desalted via stage tips and redissolved in 5% FA/5% ACN for LC–MS3 analysis.

#### Liquid Chromatography Separation and Tandem Mass Spectrometry (LC–MS3)

Proteome data were collected on an Orbitrap Fusion Lumos mass spectrometer (Thermo Fisher Scientific) coupled to a Proxeon EASY-nLC 1000 LC pump (Thermo Fisher Scientific). Fractionated peptides were separated using a 120 min gradient at 550 nL/min on a 35 cm column (i.d., 100 μm, accucore, 2.6 μm, 150 Å) packed in-house. MS1 data were collected in the Orbitrap (120,000 resolution; maximum injection time 60 ms; AGC 10 × 10^5^). Charge states between 2 and 5 were required for MS2 analysis, and a 120 s dynamic exclusion window was used. Top 10 MS2 scans were performed in the ion trap with CID fragmentation (isolation window 0.5 Da; rapid; NCE 36%; maximum injection time 50 ms; AGC 3 × 10^4^). An online real-time search algorithm (Orbiter) was used to trigger MS3 scans for quantification.^13^ MS3 scans were collected in the Orbitrap using a resolution of 50,000, NCE of 65%, maximum injection time of 200 ms, and AGC of 3.0 × 10^5^. The close out was set at two peptides per protein per fraction.^13^

### Data Analysis

Raw files were converted to mzXML, and monoisotopic peaks were reassigned using Monocle.^14^ Searches were performed using the Comet search algorithm against a *Drosophila* database downloaded from Uniprot in January 2023. A 50 ppm precursor ion tolerance, 1.0005 fragment ion tolerance, and 0.4 fragment bin offset for MS2 scans were collected in the ion trap. TMTpro on lysine residues and peptide N-termini (+304.2071 Da) and carbamidomethylation of cysteine residues (+57.0215 Da) were set as static modifications, while oxidation of methionine residues (+15.9949 Da) was set as a variable modification.

Each run was filtered separately to 1% false discovery rate (FDR) at the peptide-spectrum match level. Then, proteins were filtered to the target 1% FDR level across the entire combined data set. For reporter ion quantification, a 0.003 Da window around the theoretical *m*/*z* of each reporter ion was scanned and the most intense *m*/*z* was used. Reporter ion intensities were adjusted to correct for isotopic impurities of the different TMTpro reagents according to the manufacturer specifications. Peptides were filtered to include only those with a summed signal-to-noise (SN) of ≥180 across all TMT channels. The S/N measurements of peptides assigned to each protein were summed (for a given protein). These values were normalized so that the sum of the signal for all proteins in each channel was equivalent, thereby accounting for equal protein loading.

### Metabolic Network Analysis Using ΔFBA Approach

To investigate the metabolic gene coverage of the proteome data, gene annotations were converted to FlyBase gene IDs using the FlyBase^15^ and UniProt^16^ databases. After ensuring that the data set covers a high number of common genes with the *i*Drosophila1 genome-scale metabolic model (8230 reactions, 6990 metabolites, and 2388 genes),^17^ differential proteomic analysis was performed using the R (version 4.3.0) *limma-trend* function under the limma package by setting robust = TRUE.^18^ The fold change values of the differentially abundant proteins (*P*-value <0.05) with multiple measurements were filtered according to the criteria specified in^17^ to assign a single fold change value to each protein. The filtered fold changes were subsequently mapped to the *i*Drosophila1 reactions using the COBRA toolbox function *mapExpressionToReactions*(19) to determine differential reaction expressions. The flux of the nongrowth-associated ATP maintenance (NGAM) reaction was assumed to be unchanged relative to the control while the remaining boundaries were not constrained. Then, the differential model reactions were identified using the ΔFBA approach with a flexible threshold value (ε = 0.5) by maximizing the consistency and minimizing the inconsistency between the flux changes (Δ*v*) and differential proteomic levels.^20^ The genes and metabolites involved in these reactions were listed.

### Reaction Activity Analysis

Another network-based analysis approach was also performed to determine the differential reactions in the GenX-exposed flies. To do so, sample-based condition-specific metabolic models were first reconstructed. In this process, small flux boundaries (i.e., 10^–3^ h^–1^ and 10^–4^ mmol g^–1^ h^–1^) were set for the minimum biomass formation rate and the minimum uptake rates of glucose and oxygen to keep these reactions in the models. The minimum flux boundary of the NGAM reaction was allowed to be reduced by 50% of its experimentally detected value (8.55 mmol of ATP g^–1^ h^–1^). The duplicate measurements in the normalized proteomics data set were filtered by selecting the measurement with maximum average value across the samples for each protein. The filtered protein abundances were mapped to the *i*Drosophila1 reactions by the *mapExpressionToReactions* function. Using the integrative metabolic analysis tool (iMAT) algorithm,^21^ sample-specific metabolic network models were reconstructed for control and GenX treatment groups (in total 6 models; 3 for each group). In the reconstruction process, 25th and 75th percentiles of the whole proteome data were used as the lower and upper threshold values in the iMAT algorithm to detect active and inactive reactions based on the levels of corresponding proteins. Given the two threshold values, iMAT runs an optimization algorithm to keep reactions associated with high-level proteins in models while removing the reactions associated with low-level proteins, subject to mass-balance constraints around intracellular metabolites^22^ Based on the reconstructed sample-specific metabolic networks, differential reaction activities were identified for GenX versus control comparisons, as detailed elsewhere.^23^ Here, binary vectors were created for each sample-specific metabolic model by assigning “1” for the active (kept) reactions and “0” for the inactive (removed) reactions in the control and GenX models. If a reaction is active in all three replicates of GenX models and inactive in all three control models (or vice versa), this reaction was accepted to be significantly affected and termed differential reaction. The genes and metabolites in the differential reactions were listed. The binary vectors were also used to investigate the separation of GenX and control groups. To this aim, principal component analysis (PCA) was used with the *prcomp* function of R based on the binary vector representations of the sample-specific metabolic models. Reactions classified as inactive in all of the samples were removed prior to PCA.

### Investigation of Enriched Biological Processes and Metabolic Pathways

The GenX-mediated significantly regulated genes and metabolites, which were identified with ΔFBA and iMAT-based reaction activity analysis as described above, were merged to combine the predictive strength of these methods. The merged gene lists were analyzed in terms of significantly enriched gene-ontology (GO) biological processes and KEGG pathways for FDR < 0.05 via the recent *Drosophila*-specific tool, PANGEA (Pathway, Network and Gene-set Enrichment Analysis).^24^ Similarly, the metabolites involved in the combined metabolite lists were characterized in terms of significantly enriched KEGG pathways. To do so, KEGG IDs were first mapped to these metabolites, and they were then used as the inputs of the enrichment analysis tool under MBROLE3 (Metabolites Biological Role).^25^ In this way, significantly enriched KEGG pathways (FDR < 0.05) with at least four overlapping metabolites were identified across *Homo sapiens*. Together, the enriched terms obtained for GenX treatment were listed for both regulated genes and metabolites. In the scope of this work, MATLAB R2019a was used in the metabolic network-based analyses unless otherwise stated.

### Cell Culture and Primary Culture

#### Mixed Glial Preparation and Astrocyte Culture

Primary mixed glial cultures were isolated from postnatal day 3 (P3) to P5 mice sourced from timed pregnant C57Bl6J dams from The Jackson Laboratories, following previously outlined procedures.^26^ In short, meninges were removed and dissected cerebral cortices were dissociated and cultured in DMEM (catalog #11960-044, Thermo Fisher) supplemented with 1 mm sodium pyruvate (catalog #11360070, Corning), 20 mm HEPES (catalog #15630-080, Thermo Fisher), 100 U/mL penicillin–streptomycin (PenStrep; catalog #15140122, Thermo Fisher Scientific), 10% fetal bovine serum (catalog #S11150, R&D Systems), and 5 μg/mL insulin. Sequential immunopanning, conducted at 10 and 16 days in culture, utilized agitation at 225 rpm on an orbital shaker (model Innova 2000, New Brunswick Scientific) to separate astrocyte, microglia, and oligodendrocyte progenitor cells.

#### Astrocyte Treatments

After 2–3 days in culture following separation procedures, astrocytes were plated at a density of 150,000 cells/well on poly-l-lysine-coated 12 well plate before treatment with or without GenX (0, 1, 10, or 30 ppm) added to serum-free Neurobasal A medium containing 1% N2, 2% B27, PenStrep, 1 mm sodium pyruvate, 0.45% glucose, 5% bovine serum albumin, and 50 μm β-mercaptoethanol for 24 h.

#### Seahorse Mitostress Test

The seahorse mitostress test was performed using manufacturer’s protocol and previously published studies.^27,28^ Briefly, U373 cells were plated in a 96-well plate seahorse plate (20,000 cells/well) and treated with GenX (1 ppm) for 24 h. The Seahorse mitostress test was performed using a Seahorse Xfe96 bioanalyzer. For the mitostress test, 0.75 μM oligomycin, 1 μM FCCP, and 1 μM Rot/AA were used.

#### RNA Isolation and qPCR

To evaluate gene expression in primary murine astrocytes, total RNA was extracted using Trizol according to the manufacturer’s instructions. Subsequently, the RNA underwent quantitative real-time PCR (qRT-PCR) using either BioRad iTaq Universal Probes or primers from Integrated DNA Technologies in conjunction with SYBR Green One-Step Kits, as detailed previously^26^ using the oligonucleotides listed in Table 1.

**Table 1:** Oligonucleotides

gene name	company	sequences/catalog number
IL-1β	IDT	ATGATGGCTTATTACAGTGGCAA/GTCGGAGATTCGTAGCTGGA
IL-6	IDT	ACTCACCTCTTCAGAACGAATTG/CCATCTTTGGAAGGTTCAGGTTG
C3	IDT	GGGGAGTCCCATGTACTCTATC/GGAAGTCGTGGACAGTAACAG
Serping 1	IDT	CTGGCTGGGGATAGAGCCT/GAGATAACTGTTGTTGCGACCT
Sod1	Qiagen	#QT01671551
Gpx1	Qiagen	#QT00203392
GAPDH	IDT	ACAACTTTGGTATCGTGGAAGG/GCCATCACGCCACAGTTTC

## Results and Discussion

### GenX Exposure Leads to an Age-Dependent Reduction in Motor Behavior in Flies

**Figure 1:**
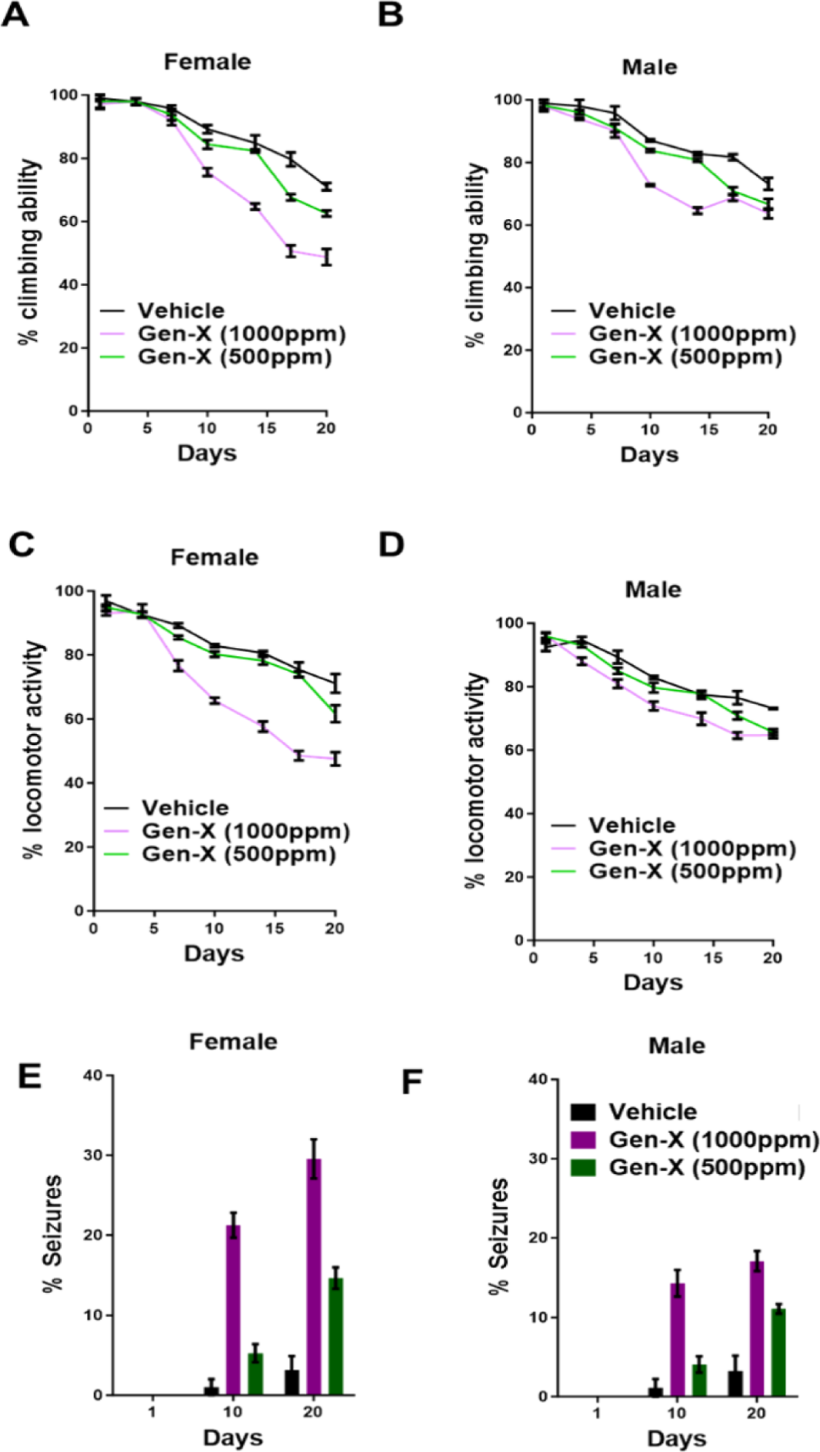
GenX exposure leads to an age-dependent reduction in motor behavior in flies. (A) Dose-dependent decreases in climbing activity in female flies exposed to 500 and 1000 ppm of GenX over 20 days. (B) Dose-dependent decreases in climbing activity in male flies exposed to 500 and 1000 ppm of GenX over 20 days. (C) Locomotor activity assay for female flies exposed to GenX at two different doses, 1000 and 500 ppm. (D) Locomotor activity assay for male flies exposed to GenX at two different doses, 1000 and 500 ppm. (E) Percentage of female flies exposed to different doses of GenX that are experiencing seizure like phenotypes over a 20-day period. (F) Percentage of male flies exposed to different doses of GenX that are experiencing seizure-like phenotypes over a 20 day period.

Exposure to GenX has been shown to be involved in reproductive, liver, and immune defects in various vertebrate models.^29−34^ Recent studies in the zebrafish model have implicated that GenX exposure can lead to probable neurotoxicity and neurotransmitter loss.^35,36^ Furthermore, a study by Vu et al. demonstrated that exposure to GenX in *Drosophila melanogaster* leads to locomotor deficits and changes in its transcriptome. However, these transcriptomic changes were not widespread.^8^ Further, this study did not look at any proteins involved in GenX toxicity or perform any cell type-specific studies. To further develop the *Drosophila* model and use this as a genetic tool, we exposed adult flies to 500 and 1000 ppm of GenX. GenX was mixed in the food of the adult flies. Since GenX was found not to accumulate in flies, we changed the food every 3 days.^8^ Unlike larvae, adult flies do not consume a lot of food. We have seen with other chemicals that the body burden of chemicals is generally 1000–5000 times less than concentrations in the food. We performed both the climbing assay^37^ and the locomotor activity assay^10^ every 3 days for 20 days. As seen in Figure 1A,C, female flies exposed to GenX demonstrate age- and dose-dependent decreases in climbing and locomotor activity deficits. However, unlike the females, male flies only showed climbing deficits at a couple of time points (days 10 and 13) (Figure 1C) and no changes in locomotor activity (Figure 1D). This is consistent with previous findings where GenX affects the female flies more than the males.^8^ Further, we observed that exposure to GenX leads to a seizure-like phenotype (Supporting Information, Video 1). The seizure-like activity observed was more pronounced in females (Figure 1E) compared to males (Figure 1F). Since we observed motor behavioral deficits in female fly 2 weeks post GenX exposure, we perform immunoblot analysis on the fly heads for tyrosine hydroxylase (TH). As shown in Figure S1, GenX leads to downregulation of TH, a marker for dopaminergic neurons. Taken together, these behavioral data support previous findings that GenX may preferentially affect females more and also lead to a motor dysfunction, TH neuronal loss, and seizure-like phenotype in flies.

### Quantitative Proteomics of GenX-Exposed Fly Brains Identifies Changes in Molecular Signatures in Fly Brains

**Figure 2:**
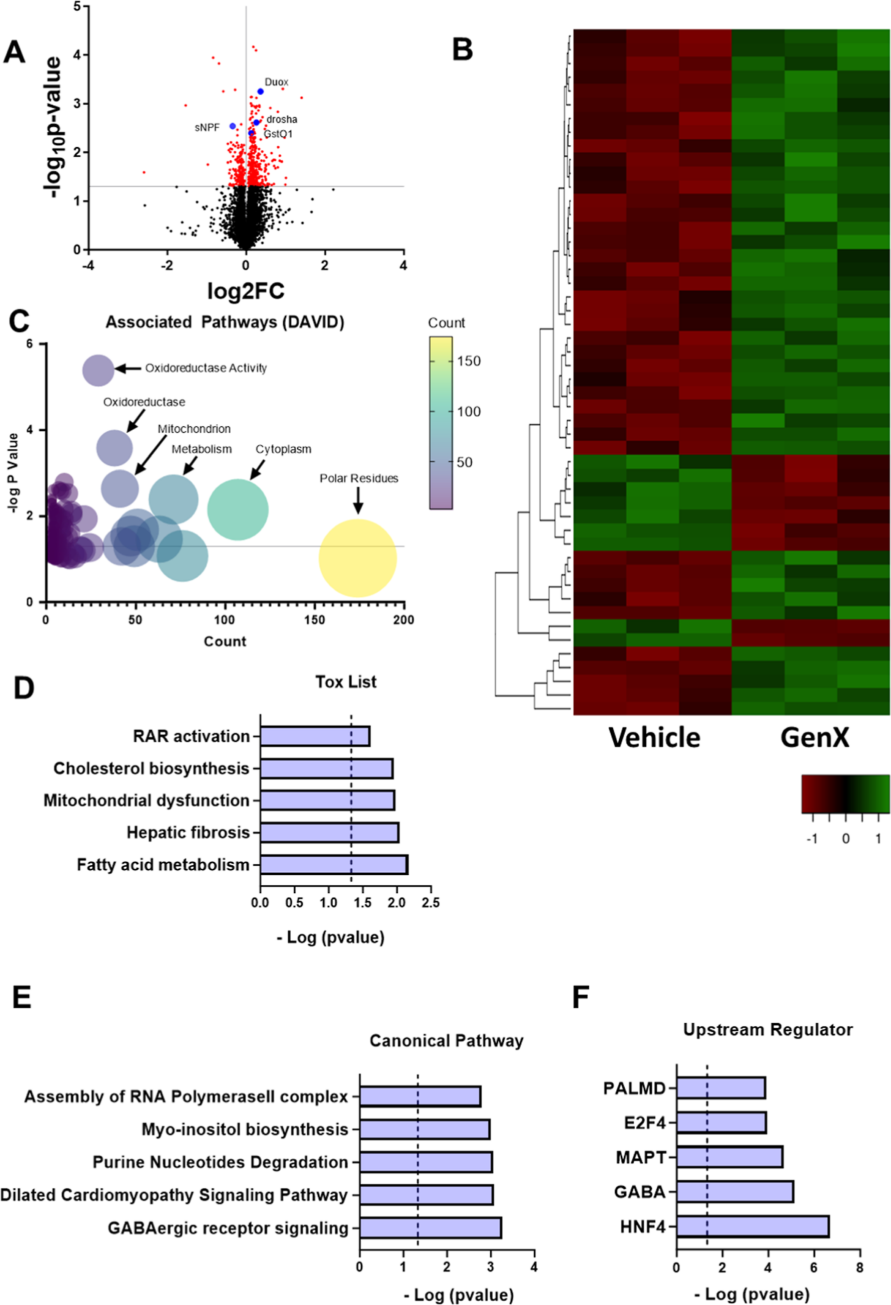
Quantitative proteomics of GenX-exposed fly brains identifies changes in molecular signatures in fly brains. (A) Volcano plot showing that protein abundance is significantly altered after 7 days of GenX exposure in female flies. (B) Heat map showing top 50 proteins that are upregulated or downregulated. (C) Enriched terms (DAVID) analysis of proteins that were affected by GenX exposure. (D) IPA showing pathways that are associated with toxic effects of the proteins. (E) IPA showing pathways commonly found altered with exposure to toxicants, that are also altered with GenX exposure. (F) IPA showing upstream regulators of the proteins that are altered post GenX exposure.

We wanted to analyze early changes in the fly brain post GenX exposure and hence performed proteomics in the fly head after exposing the flies for 5 days. At this time point, there were negligible behavioral deficits. Since female flies had a more robust response in our behavioral end points, we performed proteomics only on the female flies. Since we performed the proteomics at a time point before behavioral changes were observed and to avoid missing small changes, we took into account all proteins that were significantly altered. Approximately 517 proteins were significantly altered with 169 proteins downregulated and 348 proteins upregulated (Figure 2A). Among the top 50 proteins that are altered (Figure 2B), proteins such as Duox, sNPF, Drosha, and GstO1 are upregulated. While GstO1 is induced in response to oxidative stress, Duox has been shown to induce oxidative damage, suggesting the potential role of oxidative stress in GenX exposure. Drosha has been shown to play a critical role in driving oxidative stress and neuronal survival in dopaminergic neurons.^38^ sNPF on the other hand also has been shown to alter immune response and oxidative stress in flies.^39^ We performed DAVID analysis of the proteins that were altered by GenX exposure. As depicted in Figure 2C, GenX exposure altered metabolism, mitochondria, and oxidation–reduction processes, among others. Mitochondrial dysfunction and oxidative stress have been shown to increase when exposed to legacy PFASs.^40^ To further understand the pathways that GenX is regulating in flies, we performed the ingenuity pathway analysis (IPA).^41^ The toxicology list and functions in IPA highlight molecules, genes, and pathways that regulate a certain type of toxicity. The Tox list from IPA analysis demonstrates that the effects of GenX on the fly head are similar to mitochondrial toxicants and can regulate fatty acid metabolism and cholesterol biosynthesis (Figure 2D). Dysregulation of lipids and cholesterol has been implicated in the human population with high PFAS exposure.^42^ Further, a recent study in a population that was exposed to GenX through drinking water in North Carolina showed that GenX exposure leads to increased cholesterol.^43^ Further, pathway analysis (Figure 2E) of the proteins that were altered with GenX identified γ-aminobutyric acid (GABA) receptor signaling as a key pathway altered. GABA is the primary inhibitory neurotransmitter in the brain. GABA alteration has been implicated in epilepsy and seizures.^44^ This further validates our behavioral findings that GenX induces seizure-like activity in flies. Finally, IPA also identified GABA as a key upstream regulator of the proteins that were altered via post-GenX exposure, further suggesting that GABA may play a critical role in GenX-induced toxicity (Figure 2F). Another key upstream regulator identified by IPA was MAPT or Tau. Tau has also been implicated in various neurological disorders, including PD and Alzheimer’s disease (AD), among others. Tau has also been implicated in GABAergic neuron death suggesting a potential crosstalk between the two.^45^

### Metabolic Network-Based Analyses Show the Potential Link between GenX Exposure and Neurodegeneration in *Drosophila*

**Figure 3:**
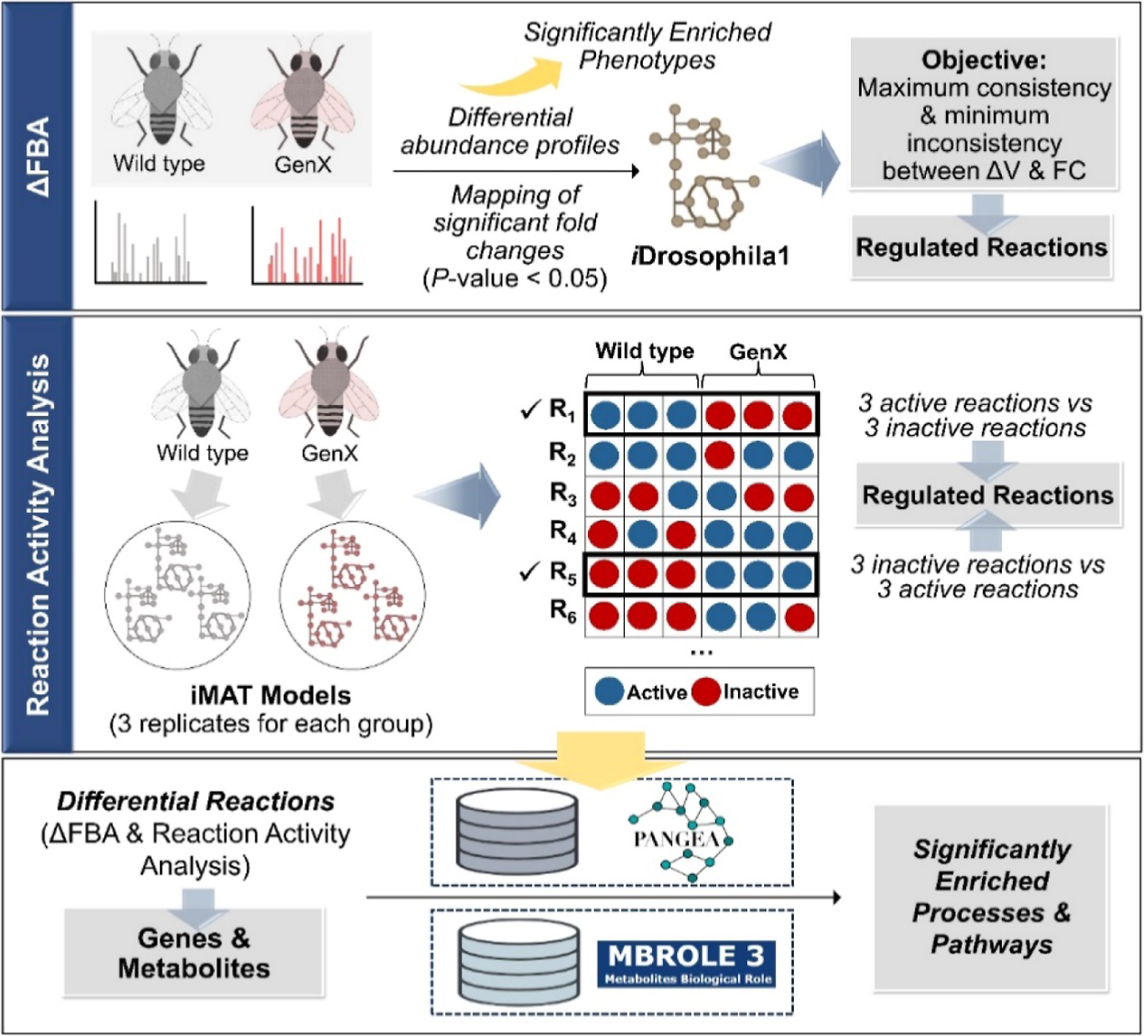
General flowchart of the metabolic network analyses. First approach is based on *in silico* identification of GenX-induced fly phenotypes with PANGEA tool and regulated reactions with ΔFBA using the differentially expressed proteins. Second approach is iMAT-based reaction activity analysis applied for extending the list of ΔFBA-predicted differential reactions. The genes and metabolites in the combined list of the differential reactions are characterized in terms of enriched terms.

**Figure 4:**
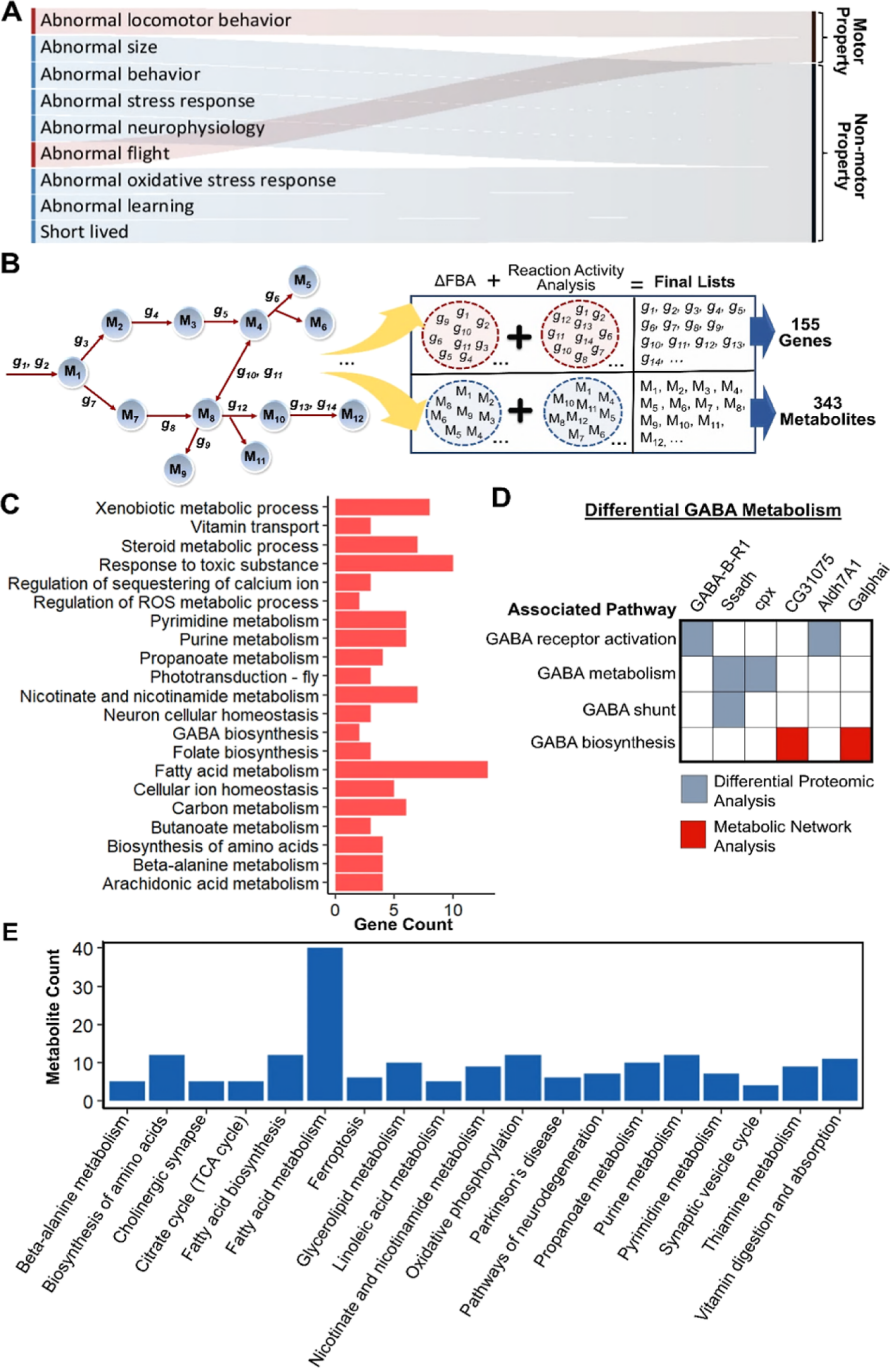
Metabolic network-based analyses of the differential metabolism induced by GenX. (A) The analysis of differentially abundant proteins (*P*-value <0.05) revealed the modulated fly phenotypes (motor and nonmotor properties). (B) Using network-based analyses, the genes and metabolites in differential reactions were determined for the GenX treatment. In this process, gene and metabolite lists were first created using two independent methods (ΔFBA and iMAT-based reaction activity analysis). Then, the lists derived from either method were combined to obtain the final regulated gene and metabolite lists, as in the simplified illustration. The final lists including a total of 155 genes and 343 metabolites were characterized in the next analyses. (C) Enrichment analysis of the GenX-induced regulated genes determined significantly enriched biological processes and metabolic pathways (FDR < 0.05). The enriched prominent terms are displayed by the bar plot and the bar sizes are proportional to the number of genes in each term. (D) GABA-related regulated pathways and the associated genes derived from the differential proteomic analysis and network-based analyses. The genes derived from each method are labeled by red and blue colors. (E) Enrichment analysis of the regulated metabolites in response to the GenX treatment was also applied to identify the over-represented metabolic pathways (FDR < 0.05). The enriched prominent terms are illustrated, and the bar sizes of the plot are proportional to the number of metabolites in each term.

In addition to the omics data-based analyses, we used metabolic network-based approaches to investigate differential metabolism in the *Drosophila* brain in response to GenX treatment. First, we confirmed the impact of the differentially abundant proteins (*P*-value <0.05) on the fly phenotype by detecting the significantly enriched FlyBase phenotypes (FDR < 0.05) via the PANGEA tool. The genes encoding the differentially abundant proteins were found to be associated with several motor (abnormal locomotor behavior and abnormal flight) and nonmotor features (e.g., abnormal oxidative stress response, abnormal neurophysiology, short living, and abnormal learning) (Figures 3 and 4A). These phenotypic properties have been observed in different neurodegenerative disorders, including PD. We subsequently characterized the metabolic alterations induced by GenX via two different algorithms based on genome-scale metabolic modeling. The most recent metabolic model of *D. melanogaster*, *i*Drosophila1, was used for this purpose. The proteome data set of the 7 day-old female fly brains contain measurements for 1634 out of 2388 genes in the *i*Drosophila1 model. Two methods applied in the scope of the network analyses include (1) ΔFBA^20^ and (2) iMAT-based reaction activity analysis.^23^ The general methodology is summarized in Figure 3.

The ΔFBA and iMAT-based reaction activity analysis uncovered a total of 155 genes involved in 85 differential reactions affected by GenX treatment (Figure 4B). Since ΔFBA is a fold change-based method and a small number of metabolic genes with significant fold changes were detected for GenX exposure, this approach predicted fewer genes representing the neurotoxicity of this chemical compound. Even if the two methods had a small number of genes in common (only three genes), most of the biological processes and metabolic pathways associated with the list of identified genes were found to be common. Furthermore, the use of these complementary methods together was clearly shown to be beneficial. For instance, reactive oxygen species (ROS) was previously reported to be stimulated in response to the GenX treatment.^46,47^ However, we did not directly identify the over-representation of this term by the ΔFBA approach. On the other hand, “regulation of ROS metabolic process” was enriched for the genes derived from the reaction activity analysis. These genes are the *WWOX* gene encoding WW domain-containing oxidoreductase and the *fdh* gene encoding formaldehyde dehydrogenase. “Fatty acid metabolism” is another pathway affected by the GenX treatment.^48^ We identified many more genes by the ΔFBA method (eight genes) related to this term when compared to the iMAT-based reaction activity analysis (five genes). Collectively, the use of both methods together is superior to expanding the gene list of interest associated with the GenX-mediated cellular changes. Therefore, the merged gene (Table S1) or metabolite lists (Table S2) involved in the differential reactions from either method were used in the rest of the study to accurately uncover the enriched terms.

For the gene-based network analyses, all enriched biological processes and metabolic pathways are listed in Table S3. The GenX exposure was shown to cause the over-representation of the pathways metabolizing essential biomolecules (e.g., “carbon metabolism”, “fatty acid metabolism”, and “purine metabolism”, among others) (Figure 4C). In previous studies, the impact of GenX treatment on the metabolomic composition of maternal and offspring liver and/or serum was investigated.^49,50^ Additionally, GenX and its novel analogs were shown to disrupt fatty acid metabolism in mice.^48^ GenX exposure affected vitamin metabolism. Accordingly, “vitamin transport”, “folate (vitamin B_9_) biosynthesis”, and “nicotinate (vitamin B_3_) and nicotinamide metabolism” were shown to be over-represented (Figure 4C). Of these vitamins, vitamin B_9_ is involved in *de novo* nucleotide synthesis^51^ and its level was reported to be reduced in neurodegenerative disorders including PD, AD, and Huntington’s disease.^52,53^ Since vitamin B_9_ deficiency leads to an increase in homocysteine level and accompanying oxidative stress, this vitamin was shown to improve the αSyn-induced locomotor defects in PD.^51,54^ Furthermore, the promising role of vitamin B_3_ in the treatment of several neurodegenerative disorders was also reported.^55,56^

We also identified the regulation of ROS as an enriched term in GenX-exposed fly brains (Figure 4C). A recent study in zebrafish also identified ROS as a regulatory factor in driving GenX toxicity.^47^ Importantly, oxidative stress arising due to mitochondrial dysfunction and abnormal ROS production has been linked to the progression of many neurodegenerative disorders.^57−60^ In addition, there is an interplay between intracellular calcium and ROS signaling systems. Therefore, a great number of pathways containing ROS-generating enzymes can be modulated by the calcium signal, and any defects in this crosstalk can lead to disorders such as AD and PD.^61^ Taken together, oxidative-stress-associated differential pathways may provide a link between GenX exposure and neurodegeneration by confirming the enriched FlyBase phenotypes in Figure 4A.

In a recent study, the developmental neurotoxicity of GenX was investigated. Wasel et al. reported an altered locomotor activity and an increased dopamine (DA) level in zebrafish^62^ in contrast to the findings of a similar, previously reported study.^63^ Rericha and colleagues also demonstrated neurodevelopmental toxicity of GenX in the zebrafish.^64^ In this organism, the GenX-mediated downregulation of *gad1b* gene encoding glutamate decarboxylase, which catalyze the conversion of glutamate to GABA, was shown.^65^ Based on the differential proteomic analysis, glutamate decarboxylase (*gad1*) was not significantly changed at the protein level. On the other hand, we revealed the over-representation of “GABA metabolism” for the GenX exposure, which is consistent with the omics data-based analysis in the previous section (Figure 4C). In accordance with this enriched term, a variety of GABA pathways were determined to have differentially abundant proteins or proteins within the GenX-mediated differential reactions (Figure 4D). The discrepancy between the findings in zebrafish and our study may stem from the time of exposure and when our proteomic study was performed. While the GenX exposure in our study were in adult flies, the exposure in the zebrafish study was developmental. Further, we performed the proteomics at a time point that preceded any motor deficits to identify early molecular signatures of GenX toxicity. In rodents, chemically stimulated locomotor activity has been shown to be reduced by the GABA_B_ receptor agonist baclofen.^66,67^ Consistently, this activity was induced in mice by GABA_B_ receptor antagonists (SCH 50911, CGP 46381, and CGP 52432) likely through the removal of GABAB receptor-mediated tonic inhibition of dopaminergic neurons and the enhancement of DA neurotransmission,^68^ despite some contradictory results in the literature for *Drosophila* due to the differences between the mammalian and *Drosophila* GABA_B_ receptors.^83,84^ It was also reported that the accumulation of extracellular GABA and the activation of GABA receptors by the inhibition of GABA reuptake transporters in adult female flies reduced locomotor activity.^85^ Similarly, Roberts et al. showed the crucial function of astrocytes in limiting the tonic GABAergic inhibition of DA release due to their GABA intake by the GABA transporters.^68,69^ We identified a significant upregulation of GABA_B_ receptor 1 (GABA-B-R1) in the GenX-treated *Drosophila* brain (Figure 4D) while TH levels are downregulated (Figure S1). Hence, the relationship of GABA receptors with dopaminergic system may contribute to several common phenotypic changes (e.g., abnormal locomotor behavior and abnormal flight) and differential metabolic patterns between GenX treatment and various neurodegenerative disorders, highlighting a need for further investigation of this circuit.

The analysis of the metabolites in the differential reactions confirmed the gene-based analysis results. For the GenX treatment, a total of 343 metabolites in the significantly affected reactions were determined by ΔFBA and iMAT-based reaction activity analysis methods (Figure 4B and Table S2). All significantly enriched KEGG pathways associated with these metabolites are represented in Table S4. Many common metabolic pathways (e.g., “biosynthesis of amino acids”, “β-alanine metabolism”, “purine metabolism”, etc.) were identified with the gene-based analysis. Of the terms enriched by the metabolite-based analysis, “citrate cycle (TCA cycle)” and “oxidative phosphorylation” are prominent (Figure 4E). The disruption of energy metabolism along with the elevated oxidative stress may be closely linked to neurodegenerative diseases and the activation of apoptotic signaling pathways. In addition, metabolite-based network analysis detected significantly over-represented terms related to neuronal synapses (“synaptic vesicle cycle”, “cholinergic synapse”, and “GABAergic synapse”). The GABAergic synapse was not included in Figure 4E because it was not significantly enriched for the selected FDR cutoff even if its *P*-value is smaller than 0.05. Thus, the enrichment results are consistent with the beforementioned potential role of GenX in the neurotransmitter modulation. While previous studies have shown the effect of PFAS and PFOS on different types of neurons, the role of GenX exposure on these nondopaminergic neurons have not been tested.^70^ Therefore, the potential effect of GenX on various neurotransmitters needs further investigation. Lastly, we determined the over-representation of “pathways of neurodegeneration–multiple diseases” and “PD” through the metabolite-based network analysis, strengthening our hypothesis on the relationship between GenX and neurodegeneration (Figure 4E). This encouraged us to comparatively characterize the differential metabolic patterns induced by human αSyn protein expressed in 7 day-old female flies. The abnormal aggregation of the αSyn proteins is a hallmark in PD.^71^ However, it has been also reported to be responsible for other similar neurodegenerative diseases, collectively called synucleopathies.^72^ The same methodology with the gene- and metabolite-based network analyses of the GenX group was also followed for the proteome data of the αSyn group. Accordingly, gene and metabolite lists in the differential reactions were first created through the proteome-guided network analyses for the 7 day-old PD model flies expressing human αSyn (Tables S5 and S6), and the associated enriched terms were uncovered. Even though the PCA plot showed the separation of the αSyn samples from the GenX and control samples in terms of the significantly affected reaction profiles (Figure S2), many common enriched biological processes or metabolic pathways associated with cellular respiration, oxidative stress, neurotransmission, and the metabolism of biomolecules were identified between the GenX and αSyn groups (Tables S7 and S8), supporting the neurotoxic potential of GenX exposure. These overlapping proteins and pathways identified provided followup targets for our further studies, enabling significant mechanistic insight into the role of GenX neurotoxicity and impaired neurobehavior in *Drosophila*.

### Comparative Proteomics and Integrating GenX with Human PD Genetics Identifies Glial Genes that are Altered by GenX Exposure

**Figure 5:**
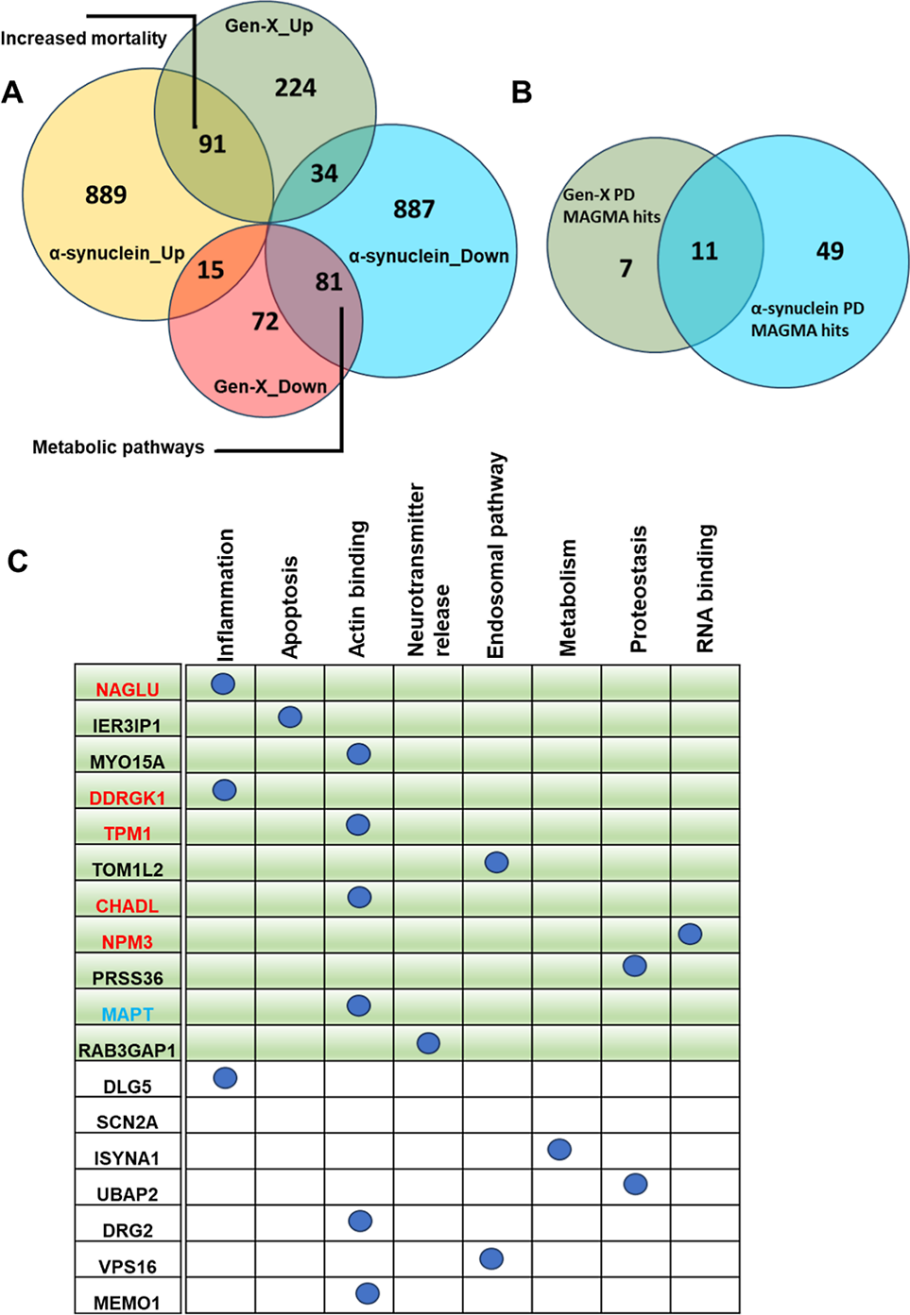
Comparative proteomics and integrating GenX with human PD genetics identifies Glial genes that are altered by GenX exposure. (A) Overlap of protein expression changes between flies exposed to GenX and those expressing human αSyn. (B) Comparing MAGMA analysis results between PD MAGMA genes in flies exposed to GenX and α synuclein, respectively. (C) MAGMA analysis results showing proteins altered in both GenX and α synuclein (highlighted in green). Highlighted in red are the proteins expressed in glia, in blue are the proteins expressed in neurons, and the proteins highlighted in black are expressed ubiquitously in all cell types.

Exposure to legacy PFAS has been shown to induce Parkinsonism in various model systems, including *Caenorhabditis elegans* and northern leopard frogs, and reduce neurotransmitter levels, including DA, among others. However, the role of second-generation PFAS in PD has not been studied. We have recently developed a genetic model of PD in *Drosophila*, where we express human αSyn in the neurons of flies. This model recapitulates various aspects of PD like age-dependent behavioral deficits,^37^ mitochondrial and metabolic defects,^73^ lysosomal dysfunction, and αSyn aggregation. We performed proteomics on 7 day-old flies that expressed human αSyn in neurons. As highlighted in the previous section, female flies were used to compare the proteomics from the αSyn flies to GenX-exposed flies. In agreement with the network-based analyses, αSyn leads to a more robust change in proteomic signatures compared to that of GenX. Interestingly, GenX and αSyn share some of the proteomic signatures. Out of the 168 proteins downregulated with GenX exposure, 81 were also downregulated in αSyn flies, while out of the 349 proteins upregulated with GenX exposure, 91 were also upregulated in αSyn flies (Figure 5A). String interaction database was used to identify enriched pathways associated with the commonly upregulated and downregulated proteins identified increased mortality and metabolism as an enriched GO term, respectively.

To further understand the relationship between the proteomic changes induced by GenX and αSyn, we performed a comparative MAGMA analysis between the two data sets. We have previously derived a comprehensive list of known human PD risk genes identified by GWAS, by performing a meta-analysis and identified 622 genetic risk factors for PD.^74^ We cross-referenced these genes to the proteins altered in the αSyn flies and GenX-exposed flies. While only 18 of the MAGMA proteins were altered in the GenX altered flies, 60 proteins were altered in the αSyn flies. Interestingly, 11 of the 18 proteins were altered with both GenX and αSyn (Figure 5B,C). Of the 11 proteins altered in both (highlighted in green in Figure 5C), five are expressed in glia (highlighted in red), five are expressed ubiquitously in all cell types (highlighted in black), and one is expressed in neurons (highlighted in blue) specifically.^75^ This data suggest that GenX may be altering proteins in the glia and not only in the neurons, suggesting a potential role of GenX in neuroinflammation. We and other researchers have shown that astrocytic and microglial inflammation plays a critical role in various neurodegenerative disorders including AD, PD, and epilepsy.^73,76−78^

### GenX Exposure Leads to Metabolic Dysregulation and Enhances Markers of A1 Astrocytes in Cell Culture

**Figure 6:**
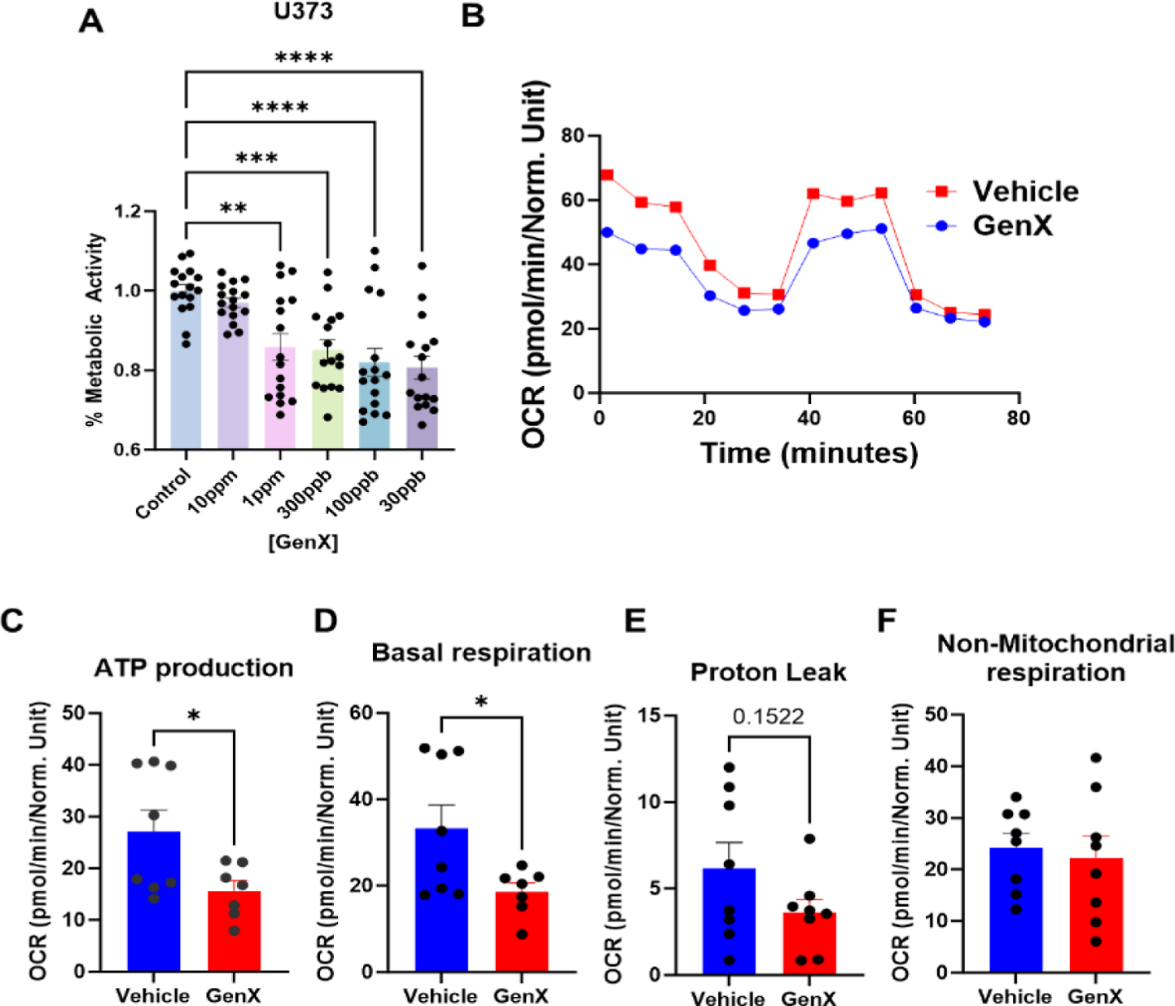
GenX exposure leads to metabolic dysregulation in cell culture. (A) MTS assay showing the change in metabolic activity of human astrocytic cell line, U373, after treatment with different doses of GenX for 24 h. (B) MitoStress test using a Seahorse Xfe96 bioanalyzer on U373 cells treated with 1 ppm GenX for 24 h. (C) The effect of treating U373 cells with 1 ppm GenX for 24 h on ATP production. (D) The effect of treating U373 cells with 1 ppm GenX for 24 h on basal respiration. (E) The effect of treating U373 cells with 1 ppm GenX for 24 h on proton leak. (F) The effect of treating U373 cells with 1 ppm GenX for 24 h on nonmitochondrial respiration. All data are analyzed using Student’s *t*-test. *N* = 3–4. *p < 0.05, **p < 0.01, ***p < 0.005.

**Figure 7:**
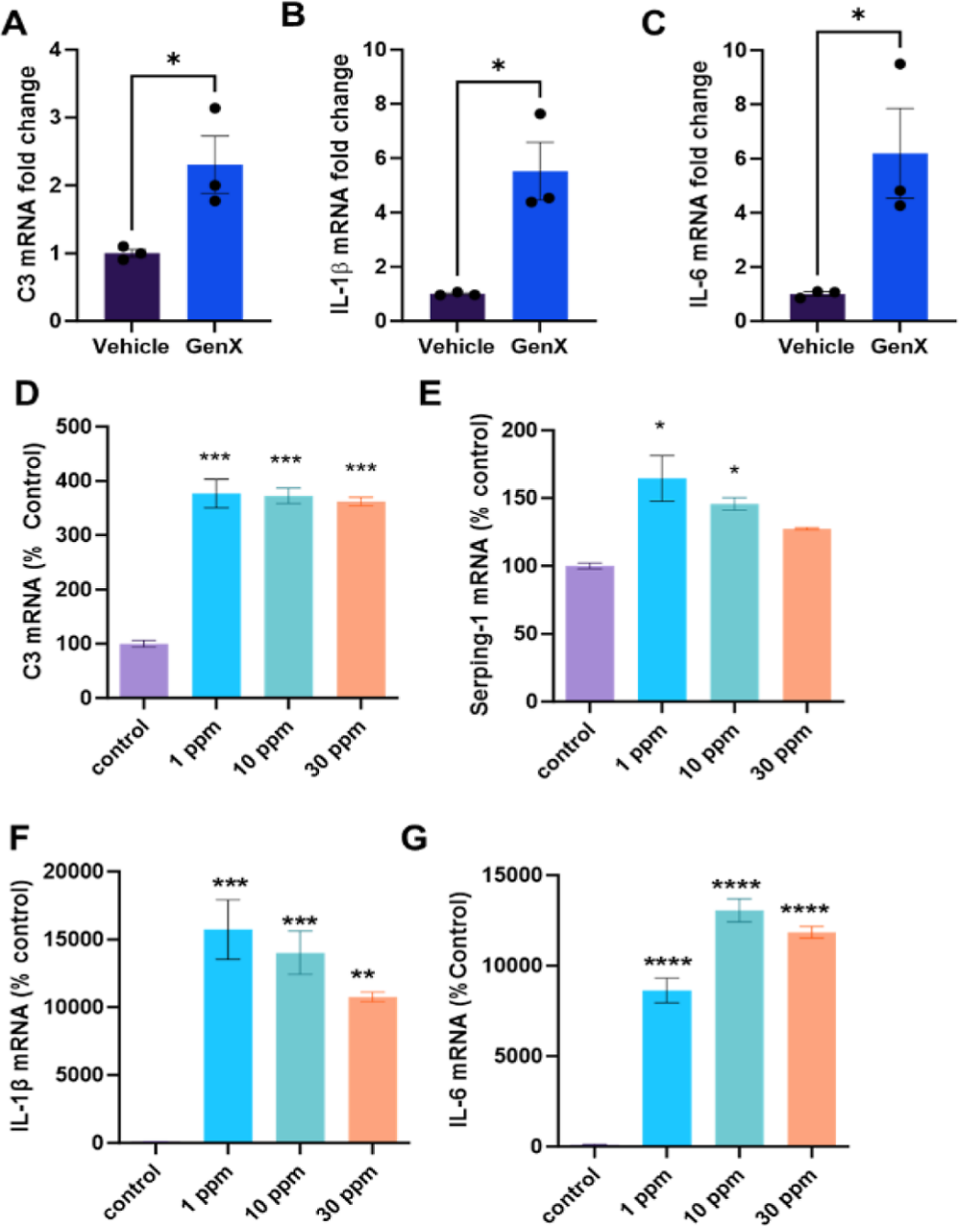
GenX exposure leads to enhanced markers of A1 astrocytes in cell culture. (A–C) qPCR analysis showing (A) C3, (B) IL-1β, and (C) IL-6 mRNA fold change in U373 astrocytes treated with 1 ppm GenX for 24 h, (D–G) qPCR analysis of mouse primary astrocytes treated with different doses of GenX for 24 h, showing change in (D) C3, (E) Serpin 1, (F) IL-1β, and (G) IL-6 mRNA levels relative to control. All data are analyzed using Student’s *t*-test. *N* = 3–4. *p < 0.05, **p < 0.01, ***p < 0.005.

Our proteomic data (Figure 2) and the *i*Drosophila1 metabolic model (Figure 4) indicated that GenX exposure might be regulating GABA. Astrocytes can produce GABA and can also regulate the levels of GABA through signaling mechanisms.^79^ The majority of the proteins identified in the MAGMA analysis are expressed exclusively in glia or ubiquitously. Hence, we concentrated on understanding the impact of GenX on astrocytes. First, we used the human astrocytic cell line, U373, treated the cells with different doses of GenX, and performed an MTS assay. As shown in Figure 6A, low doses of GenX (30 ppb to 1 ppm) led to a decrease in metabolic activity. Interestingly, a high dose of 10 ppm did not cause significant metabolic defects in astrocytes. For future studies, we used 1 ppm of GenX. Since our proteomics data indicated metabolic defects and changes in the TCA cycle, we performed a Seahorse MitoStress assay on human astrocytes treated with GenX (Figure 6B). GenX reduced mitochondrial ATP production (Figure 6C), basal respiration (Figure 6D), and proton leak (Figure 6E), while not altering nonmitochondrial respiration (Figure 6F). These data suggest that GenX leads to mitochondrial dysfunction in astrocytes. Previously, various legacy PFASs have been shown to induce mitochondrial dysfunction. We have previously demonstrated that mitochondrial dysfunction in astrocytes leads to an increase in astrocytic inflammation.^80^ Further, we have shown that mitochondrial dysfunction in astrocytes also leads to an A1-type phenotype^27^ that has been linked to astroglial aging.^81^ To understand if GenX induces an A1-like phenotype in astrocytes, we treated the human astrocytic cell line (Figure 7A–C) and mouse primary astrocytes (Figure 7D–G) with GenX. Quantitative PCR analysis revealed that the A1 markers of astrocytes, C3, Serping 1, IL-1β, and IL-6 were upregulated in astrocytes treated with GenX. Taken together, these data suggest that GenX may target astrocytes, leading to changes in mitochondrial dynamics and A1-like activation of astrocytes. A recent study has highlighted the importance of A1 astrocytes in promoting ferroptosis in epilepsy.^82^ Further research is warranted to investigate the role of astrocytic aging post GenX exposure and its role in regulating noncell autonomous neurodegeneration.

A previous study has shown that legacy PFASs lead to oxidative stress by lowering antioxidants such as superoxide dismutase 1 (Sod1).^86^ To test whether GenX also alters oxidative stress through a similar pathway, we treated the human astrocytic cell line with GenX and performed qPCR analysis for various antioxidant enzymes. As shown in Figure S3, GenX did not alter the mRNA level of Gpx1 (Figure S3A) and Sod1 (Figure. S3B). Intriguingly, a catalase activity assay demonstrated that GenX did reduce catalase activity (Figure S3C) in astrocytes, suggesting a potential role of this peroxisomal enzyme specifically and not other antioxidant factors. Further, future studies concentrating on the mechanisms of GenX-induced astroglial aging are required, concentrating on metabolism, mitochondria, and catalase-induced oxidation–reduction processes, among others.

Our studies shed light on the potential long-term effect of GenX in modulating gene expression, mitochondrial dynamics, and neurobehavior. The metabolic dysregulation shown here in *Drosophila* aligns with altered glial gene expression patterns that parallel those observed in PD models, alluding to GenX’s potential role in neuroinflammation and neurodegenerative processes. Notably, the differential impact on female flies underscores the need to further dissect sex-specific toxicological effects further. Our investigations reveal that GenX causes a complex network of disruptions, from decreased mitochondrial activity in astrocytes to the upregulation of inflammatory A1 astrocyte markers. This cascade of cellular stressors likely contributes to the neurobehavioral phenotypes observed in Figure 1, offering a glimpse into the underlying mechanisms that could be at play in humans exposed to PFAS compounds. In future studies, the role of astrocytic aging post-GenX exposure remains a key frontier, especially in its potential to mediate noncell autonomous neurodegeneration. It is our hope that these findings will spur further research, particularly into the long-term effects of environmental contaminants on neurological health and their interplay with genetic predispositions to neurodegenerative diseases.

## Supporting Information Available

Supplemental tables (XLSX)
Video 1 showing that exposure to GenX leads to a seizure-like phenotype (MOV)
GenX reduces TH levels in flies; PCA analysis of proteomics; GenX alters catalase activity but not other antioxidant enzymes in astrocytes; list of the genes and metabolites in the differential reactions obtained by metabolic network-based analyses for the GenX treatment and α-synuclein (αSyn) treatment; and significantly enriched GO biological processes and KEGG pathways (PDF)

## Supplementary Material

es4c05472_si_001.xlsx

es4c05472_si_002.mov
