# Supplementary material for: Comparative Proteomics Highlights that GenX Exposure Leads to Metabolic Defects and Inflammation in Astrocytes
Source: Environ Sci Technol. 2024 Nov 5;58(46):20525–39. doi: 10.1021/acs.est.4c05472 (PMC11580177; doi:10.1021/acs.est.4c05472)
Supplement: Supplementary file 3 — es4c05472_si_003.pdf [file es4c05472_si_003.pdf]

Comparative proteomics highlights that GenX exposure leads to metabolic defects and inflammation in astrocytes.

Abdulla Abu-Salah[1$], Müberra Fatma Cesur[2$], Aiesha Anchan[3], Muhammet Ay[1], Monica R. Langley[4], Ahmed Shah[1], Pablo Reina-Gonzalez [1], Rachel Strazdins[1],Tunahan Çakır[2], Souvarish Sarkar[1,3#]

1- Dept of Environmental Medicine, University of Rochester Medical Center, 575 Elmwood Avenue, Rochester, NY, 14620, USA.
2- Department of Bioengineering, Gebze Technical University, Gebze/KOCAELİ, 41400, Turkey.
3- Dept of Neuroscience, University of Rochester Medical Center, 575 Elmwood Avenue, Rochester, NY, 14620, USA.
4- Department of Molecular Pharmacology & Experimental Therapeutics, Department of Neurology, Department of Physical Medicine & Rehabilitation, Mayo Clinic, Gonda Building, 19th Floor, 200 First St. SW, Rochester, MN 55905, USA.

$ A.A. and M.F.C contributed equally to this paper.

# correspondence should be addressed to Dr. Souvarish Sarkar, Dept of Environmental Medicine, URMC, email:Souvarish_sarkar@urmc.rochester.edu

Number of Supplemental Figures: 3

Number of Supplemental Tables: 8

Number of Supplemental Videos: 1

Table S1:“List of the genes in the differential reactions obtained by metabolic network-based analyses for the GenX treatment. These analyses include ΔFBA and iMAT-based reaction activity analysis."

Table S2: “List of the metabolites in the differential reactions obtained by metabolic network-based analyses for GenX treatment. These analyses include ΔFBA and iMAT-based reaction activity analysis."

Table S3: “Significantly enriched GO biological processes and KEGG pathways (FDR < 0.05) of the genes in the GenX-induced differential Drosophila reactions that were determined by the PANGEA tool."

Table S4: “Significantly enriched KEGG pathways (FDR < 0.05) of the metabolites in the GenX-induced differential Drosophila reactions that were determined by the MBROLE3 server. It should be noted that the only terms including at least four overlapping metabolites are included in the table."

Table S5: “List of the genes in the differential reactions obtained by metabolic network-based analyses for the α-synuclein (αSyn) treatment. These analyses include ΔFBA and iMAT-based reaction activity analysis."

Table S6: “List of the metabolites in the differential reactions obtained by metabolic network-based analyses for the α-synuclein (αSyn) treatment. These analyses include ΔFBA and iMAT-based reaction activity analysis."

Table S7: “Significantly enriched GO biological processes and KEGG pathways (FDR < 0.05) of the genes involved in the differential Drosophila reactions. The enriched terms obtained by the PANGEA tool are comparatively represented for GenX and αSyn groups."

Table S8: “Significantly enriched KEGG pathways (FDR < 0.05) of the metabolites involved in the differential Drosophila reactions. The enriched terms obtained by the MBROLE3 server are comparatively represented for GenX and αSyn groups. It should be noted that the only terms including at least four overlapping metabolites are considered."

Supplemental Figure 1

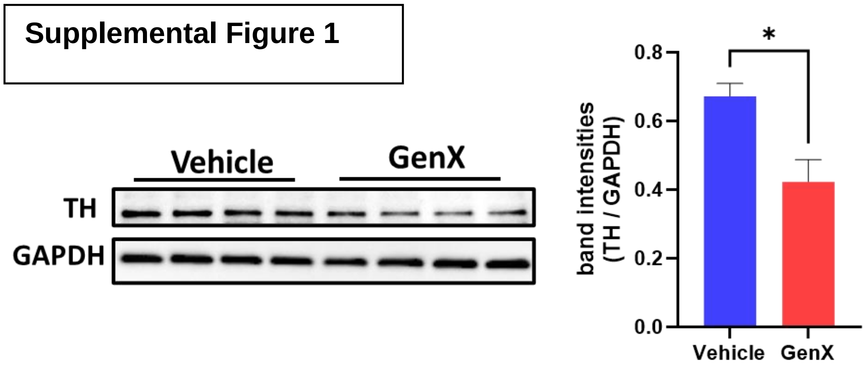

Figure S1: GenX reduces TH levels in flies: Drosophila were exposed to 1000 ppm GenX for 20 days. Western blot analysis of fly heads demonstrates reduction in TH protein levels. n=4. Data were analyzed using Stedent's t test. * $p<0.05$

Supplemental Figure 2

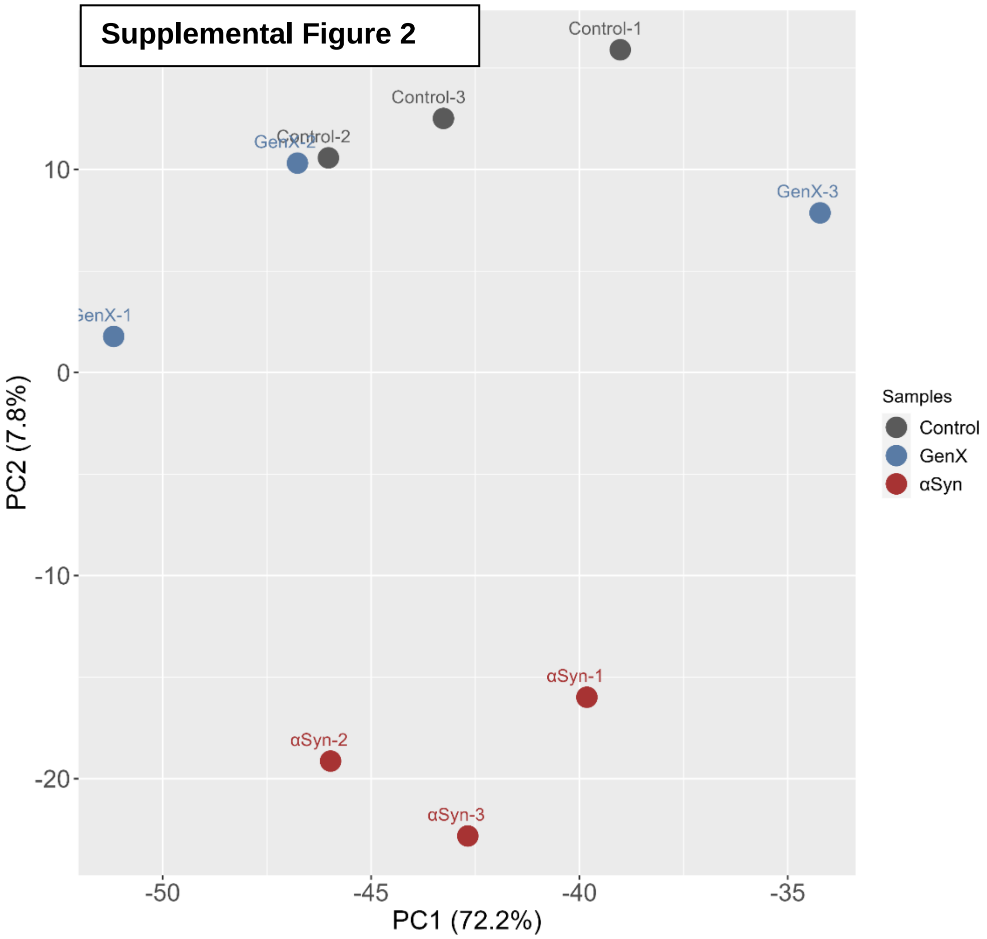

Figure S2: Reaction activity-based distribution of the αSyn, GenX, and control samples using the PCA approach.

Supplemental Figure 3

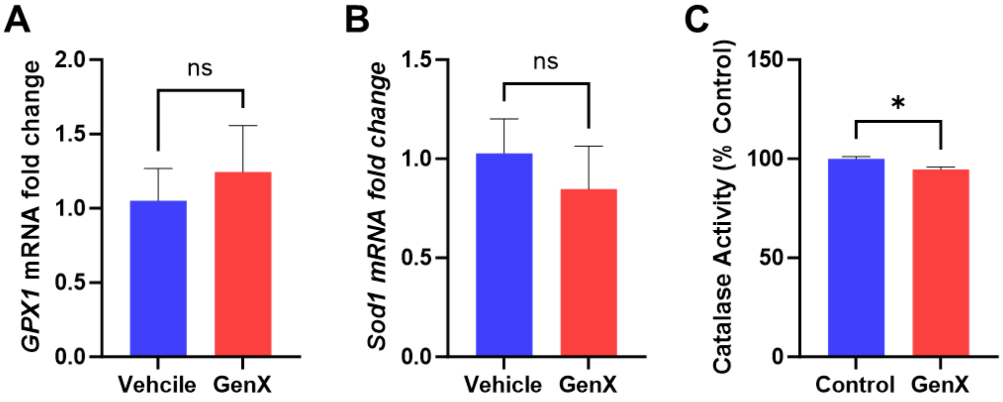

Figure S3: GenX alters catalase activity but not other antioxidant enzymes in astrocytes. U373 human astrocytic cell line were treated with 1 ppm GenX for 24 h. A-B) qPCR analysis of A) *GPX1* and B) *Sod1* mRNA levels post GenX exposure. C) Catalase activity assay demonstrates reduction in activity post GenX treatment. For A and B, n=3, for C N=5-6. Data were analyzed using Student's t test. ns- not significant, * $p<0.05$
